# Checkpoint Kinase 1 (CHK1) Functions as Both a Diagnostic Marker and a Regulator of Epithelial-to-Mesenchymal Transition (EMT) in Triple-Negative Breast Cancer

**DOI:** 10.3390/cimb44120398

**Published:** 2022-11-23

**Authors:** Hyo-Jin Kim, Bo-Gyeong Seo, Eun-Chan Seo, Kwang-Min Lee, Cheol Hwangbo

**Affiliations:** 1 Division of Life Science, College of Natural Sciences, Gyeongsang National University, Jinju 52828, Republic of Korea; 2 Division of Applied Life Science (BK21 Four), PMBBRC and Research Institute of Life Sciences, Geongsang National University, Jinju 52828, Republic of Korea

**Keywords:** TNBC, triple-negative breast cancer, Gene Expression Omnibus, DEG, differentially expressed genes, survival rate

## Abstract

Triple-negative breast cancer (TNBC) is more difficult to treat and has a higher mortality rate than other subtypes. Although hormone receptor-targeted therapy is an effective treatment to increase survival rate in breast cancer patients, it is not suitable for TNBC patients. To address the issues, differentially expressed genes (DEGs) in TNBC patients from the Gene Expression Omnibus (GEO) database were analyzed. A total of 170 genes were obtained from three Genomic Spatial Events (GSEs) using the intersection of each GSE dataset and 61 DEGs were identified after validation with the gene enrichment analysis. We combined this with the degree scores from the Kyoto Encyclopedia of Genes and Genomes (KEGG) pathway and protein-protein interaction (PPI) network, of which 7 genes were correlated with survival rate. Finally, a proteomics database revealed that only the CHK1 protein level was differently expressed in basal-like compared with other subtypes. We demonstrated that CHK1 expression was higher in TNBC cell lines compared with non-TNBC cell lines, and CHK1 promotes epithelial to mesenchymal transition (EMT) as well as migration and invasion ability. Our study provides new insight into the TNBC subnetwork that may be useful in the prognosis and treatment of TNBC patients.

## 1. Introduction

Breast cancer, the second leading cause of death, is the most frequently diagnosed cancer among women worldwide, accounting for 30% of all new cancer diagnoses in 2019 [1,2]. Although the mortality rate of breast cancer is decreasing due to the continuous development of therapies such as targeted treatment, triple-negative breast cancer (TNBC), in particular, still has a high mortality rate [3,4,5,6]. Four major molecular subtypes of breast cancer have been identified by comprehensive gene expression profiling, including luminal A, luminal B, human epidermal growth factor receptor-2 (HER2) positive, and TNBC, which are described by specific biological features, morphological patterns, and most significantly, distinct clinical processes and prognoses [7,8]. TNBC can be classified into more granular subtypes, generally divided into four or more types [9]. Studies according to subdivided TNBC are ongoing, but further research is still needed on TNBC, a higher class that shows the worst prognosis.

The most aggressive subtype of breast cancer is TNBC, which lacks expression of estrogen receptor (ER), progesterone receptor (PR), and HER2. In general, TNBC patients have a late diagnosis with poor prognosis and higher therapy resources compared with other breast cancer subtypes [10]. Due to the lack of expression of these receptors, TNBC patients are often diagnosed late with a high grade and cannot be treated with hormonal or targeted therapies, unlike hormone receptor-positive breast cancers. Therefore, various combination treatments are used for TNBC, but more research is required because there is no clear molecular target [11,12]. TP53 and BRCA1 germline mutations are discovered in TNBC patients [13,14,15]. However, the correlation between a specific gene and cancer cell metastasis is unclear [16], so excavation of the target gene is necessary for the precise diagnosis and treatment of TNBC patients.

Checkpoint kinase 1 (CHK1) is encoded by the CHEK1 gene and is a serine/threonine-specific protein kinase [17,18]. CHK1 is activated by ataxia-telangiectasia and Rad3-related (ATR) kinase via phosphorylation in response to DNA damage [19]. Activated CHK1 induces Cdc25 proteasomal degradation via phosphorylation [20,21,22]; as a result, this pathway induces cell cycle arrest [23]. Cdc25 degradation inhibits cyclin-dependent kinase (Cdk) complex formation; it can occur at multiple checkpoints including the S phase and G2/M arrest [24]. Overexpression of CHK1 has been reported in various human cancers, such as colon, breast, stomach, cervical, and liver cancer [25,26,27,28,29]. CHK1 inactivation reduces tumor growth [30,31] and chemotherapy resistance [32,33].

In this study, we sought to identify genes with irregular expression changes in TNBC by analyzing the Gene Expression Omnibus (GEO) database. We analyzed differentially expressed genes (DEGs) in TNBC compared with typical or other breast cancer subtypes in the three GSE datasets (GSE36295, GSE36693, GSE65216). Next, the biological pathway and protein-protein interaction (PPI) analysis between the selected genes in the three GEO datasets was evaluated. Through the survival rate analysis, we confirmed the correlation between DEGs and TNBC patients. In addition, we found that the CHK1 protein was meaningfully increased in TNBC compared with non-TNBC using proteome atlas. We confirmed that the expression of CHK1 was higher in TNBC cell lines than in non-TNBC cell lines. CHK1 induced epithelial marker proteins and reduced mesenchymal marker proteins. In addition, mesenchymal shape was confirmed in CHK1 expression cells. The migration and invasion activity were increased in CHK1-overexpression cells but decreased in CHK1-knockdown cells. Combining these results, we suggest a new perspective in that CHK1 can be utilized as a treatment and diagnostic target gene for TNBC patients.

## 2. Materials and Methods

### 2.1. GEO Datasets

GEO datasets from the database ‘https://www.ncbi.nlm.nih.gov/geo/’ (29 January 2021) are based on the microarray. GEO datasets were searched by some researching words: (“breast neoplasms” [MeSH Terms] OR breast cancer [All Fields]) AND “Homo sapiens” [porgn] AND “Expression profiling by array” [Filter]. Of the above search results, we selected the datasets including more than 40 samples, normal or other subtypes and TNBC results, and performed a microarray using another probe set. According to the screening criteria, three GEO datasets (GSE36295 [34], GSE36693 [35] and GSE65216 [35]) were downloaded from the GEO database. GSE36295 was based on GPL6244 (Affymetrix Human Gene 1.0 ST Array [transcript (gene) version]), GSE36693 was based on GPL10558 (Illumina HumanHT-12 V4.0 expression beadchip), and GSE65216 was based on GPL570 (Affymetrix Human Genome U133 Plus 2.0 Array). A total of 85 samples were explored in the present study, including 205 non-TNBC samples.

### 2.2. Identification of Gene Expression Data in TNBC Patients

The GEO2R data of GSE36295, GSE36693, and GSE65216 are downloaded from GEO (https://www.ncbi.nlm.nih.gov/geo/ accessed on 29 January 2021). The data classified TNBC versus normal or non-TNBC. For analysis, the normalization data was compiled from gene symbol, fold change (FC) log, and *p*-value obtained by GEO2R. The distribution of gene expression was cut-off from the iPathwayGuide site (https://advaitabio.com/ipathwayguide/ accessed on 15 November 2022) to FC log > 1, *p* < 0.01, and visualized with volcano plot [36]. The list of genes selected by the statistical cut-off was downloaded from the iPathwayGuide site. The Venn diagram showing the intersection between the three gene lists from each of the GSEs was drawn using the FunRich software (http://www.funrich.org accessed on 26 October 2020) [37]. The heatmap visualized the FC log value of total DEGs using GraphPad prism^®^ version 7.01. Up-regulated genes were marked in red and down-regulated genes in green, and the right-hand axis next to them was marked with numerical values according to color concentration.

### 2.3. Gene Enrichment Analysis

Gene enrichment was analyzed and visualized in each up-regulated and down-regulated DEG group using FunRich software. Each DEG file was added and analyzed in the ‘gene enrichment’ section to classify the ‘biological pathway’, ‘cellular component’, and ‘molecular function’ of the genes.

### 2.4. Protein-Protein Interation (PPI) Network Analysis

PPI network analysis and visualization were performed using the STRING website (https://string-db.org/ accessed on 29 September 2022). A list file of up-regulated and down-regulated DEGs was uploaded to the website, obtaining results of the PPI network and KEGG pathway. Visualization of network analysis was performed using Cytoscape software (version 3.8.1) and MCODE plug-in (version 2.0.2).

### 2.5. Survival Rate Analysis

The survival rate of breast cancer patients was obtained from the Kaplan-Meier (KM) plotter website (https://kmplot.com/analysis/ accessed on 27 January 2022). In the breast cancer section, ER, PR and HER2 status were all set to negative and the correlation between selected gene expression or protein expression and survival rates was analyzed.

### 2.6. Cell Culture

MCF-7, MDA-MB231, T47D, MDA-MB453, BT549, and Hs578t cells were purchased from American Type Culture Collection (ATCC). MCF-7, MDA-MB231, T47D, MDA-MB453, and Hs579t cells were cultured in Dulbecco′s Modified Eagle′s Medium (DMEM, Sigma, St. Louis, MO, USA, D6429) supplemented with 10% Fetal Bovine Serum (FBS, Merck, Darmstadt, Germany, TMS-013-BKR) and 1% Penicillin-Streptomycin (Lonza, Basel, Switzerland, 17-602E). BT549 cells were cultured in Roswell Park Memorial Institute (RPMI) 1640 Medium (Sigma, R8758) supplemented with 10% Fetal Bovine Serum (FBS, Merck, TMS-013-BKR) and 1% Penicillin-Streptomycin (Lonza, 17-602E). Cells were maintained in a humidified 5% CO_2_ incubator at 37 °C.

### 2.7. Reverse Transcription-PCR Analysis

The RNA of MCF-7 and MDA-MB231 cells was obtained using the RNeasy mini kit (Qiagen, Valencia, CA, USA) according to the manufacturer’s instructions. The complementary DNA (cDNA) was synthesized using the iScript cDNA Synthesis Kit (Bio-rad, Hercules, CA, USA, BR-170-8891). RT-PCR was conducted with cDNA and specific gene primers using IQ SYBR Green Supermix (Bio-rad, BR1708882). Primer sequence is represented in the following Table 1. mRNA expression level was quantified using the Graphpad program.

### 2.8. Protein Expression Analysis

Protein expression comparison between basal-like and HER2-positive, luminal A, and luminal B breast cancer subtypes were analyzed from the cancer proteome atlas (https://www.tcpaportal.org/tcpa/ accessed on 11 March 2022).

### 2.9. DNA and siRNA Transfection and Western Blotting

The cDNA of CHK1 was purchased from Korea Human Gene Bank (hMU012160). cDNA of CHK1 was transferred to the pCMV-HA plasmid purchased from Addgene. DNA transfection in MCF-7 cells was performed using Lipofectamin 3000 (Invitrogen, Waltham, MA, USA, L3000015) according to the manufacturer’s instructions. Briefly, Lipofectamin 3000 and DNA with p3000 reagent were diluted in DMEM media, respectively. We blended the above mixtures and incubated for 15 min. The mixture was treated in MCF-7 cells for 6 h; the medium was exchanged with a complete DMEM medium. AccuTarget™ Negative Control siRNA (SN-1002) as a control of siRNA was purchased from Bioneer. The siRNA of CHK1 was purchased from Invitrogen (HSS101854). siRNA transfection in MDA-MB231 cells was performed using Lipofectamin RNAiMAX (Invitrogen, 13778-150) according to the manufacturer’s instructions. Briefly, siRNA and RNAiMAX reagent were mixed in free media and incubated for 15 min. The mixture was treated in MDA-MB231 cells for 6 h; the medium was exchanged with a complete DMEM medium. After transfection of 48 h, cells were washed in phosphate-buffered saline (PBS) and lysed in lysis buffer (50 mM Tris (pH7.4), 150 nM NaCl, 1% NP-40 and 1 mM EDTA, protease/phosphatase inhibitors cocktail (Cell signaling, Danvers, MA, USA, 5872S)) for 30 min in ice. It was centrifuged at 15,000 rpm at 4 °C for 30 min, and the cell lysate was loaded onto 8–12% SDS-PAGE. Subsequently, it was transferred to a polyvinylidene difluoride membrane (Bio-rad, 1620177) and then blocked with 5% blocking reagent (Genomicbase, Seoul, Korea, SKI400). This membrane reacted with appropriate primary antibodies; antibodies for CHEK1 (sc-8408) and β-actin (sc-47778) were purchased from Santa Cruz. E-cadherin (610181) and N-cadherin (610920) antibodies were purchased from BD Biosciences. Occludin (40-4700) was purchased form Invitrogen and Vimentin (5741s) was purchased from Cell signaling technology. For protein visualization, we bound a suitable secondary antibody for 1 h and chemiluminescence signals were detected by the Clarity ECL substrate Kit (Bio-rad, 1705061).

### 2.10. Phalloidin Immunofluorescence

Cells were washed in PBS and fixed with 4% formaldehyde (Sigma, SHGJ2885) diluted in PBS for 10 min in RT. After PBS washing, the permeabilization of cells was performed with 0.1% IGEPAL (Sigma, I3021) diluted by 5% BSA (Bioshop, Burlington, Canada, ALB001.100) in PBS for 1 h in RT. After PBS washing, cells were stained with Phalloidin antibody (Invitrogen, A12380) diluted by 5% BSA in PBS for 2 h at 4 °C. After incubation, cells were washed in PBS and nuclei stained with DAPI (Invitrogen, D1306) for 10 min in RT. Fluorescence microscopy was performed using Olympus BX-UCB (Olympus, Tokyo, Japan).

### 2.11. Wound Healing Assay

MCF-7 and MDA-MB231 cells were seeded in a 6-well plate (SPL, 30006) and transfected DNA or siRNA according to 2.9. After transfection, cells were incubated until they reached 90% confluency and a linear scratch was made using a 200 µL sterile pipette tip. The image was obtained from microscopy at a described time. The wound area was quantitated by imageJ (version 1.52a) and presented by GraphPad prism^®^ version 7.04.

### 2.12. Transwell Invasion Assay

Transwell invasion assays were conducted using the Boyden chamber method and polycarbonate membranes with an 8-μm pore size (Falcon, New York, NY, USA, 353097). First, fibronectin (Corning, New York, NY, USA, 354008) is coated outside of the membrane at a concentration of 100 μg/mL for 1 h. The inside of the membrane was coated with 10% diluted Matrigel^®^ (Corning, 354234) in PBS for 1 h. DNA- or siRNA-transfected cells, according to 2.9, were seeded in triplicate at a density of 2.5 × 10^4^ cells into the chamber with 200 µL serum-free DMEM, and DMEM containing 10% FBS was placed into the lower chambers. After MCF-7 and MDA-MB231 cells were incubated for 48 h and 12 h in humidified 5% CO_2_ in a 37 °C incubator, respectively, invaded cells on the lower surface of the membrane filter were fixed with 4% formaldehyde (Sigma, SHGJ2885) diluted in PBS. After PBS washing, the membrane was stained with DAPI (Invitrogen, D1306). The image was obtained using Olympus BX-UCB. Then the cell counted at least five randomly selected microscopic fields (×20) per filter.

### 2.13. Statistics

Statistical analysis was performed using the GraphPad prism^®^ version 7.04. For pair comparisons, data were tested by one-way analysis of variance (ANOVA). The *t*-test was applied for multiple comparisons. Graphs present the means of SEM. All data were repeated in triplicate.

## 3. Results

### 3.1. Identification and Clustering of DEGs in TNBC

**Figure 1:**
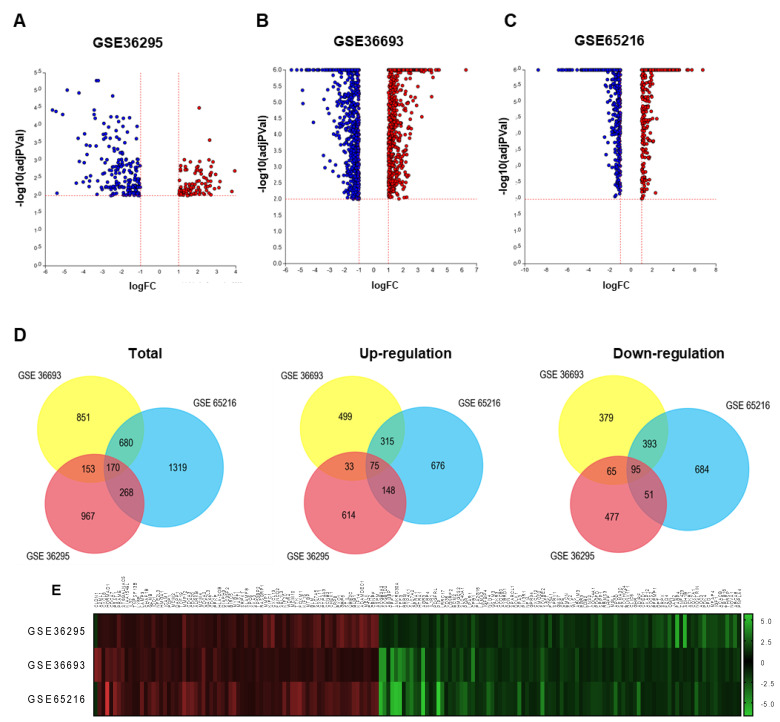
Gene expression pattern with each GSE dataset and clustering of DEGs heat map of overlapping genes. (**A**) The volcano plot shows gene expression distribution of the microarray data in GSE36295, (**B**) GSE36693, and (**C**) GSE65216. The data was cut-off based on *p*-value < 0.01 and fold change (FC) log > 1. X-axis and y-axis present fold change log and log-transformed *p*-value, respectively. (**D**) Venn diagram for intersection of all up-regulated and down-regulated DEGs of GSE36295, GSE36693, and GSE65216 using the FunRich program. (**E**) Heat map exhibiting expression changed genes of up-regulated and down-regulated DEGs using GraphPad.

We obtained DEGs from three gene expression profiles (GSE36295, GSE36693, and GSE65216) selected in this study (Table 2). Based on the criteria of *p* < 0.01 and fold change (FC) log > 1, 1556 DEGs were identified from the profiles based on GPL6244, including 869 up-regulated genes and 687 down-regulated genes (Figure 1A); 1852 DEGs were identified from the profiles based on GPL10558, including 921 up-regulated genes and 931 down-regulated genes (Figure 1B); 2434 DEGs were identified from the profiles based on GPL570, including 1213 up-regulated genes and 1221 down-regulated genes (Figure 1C). A total of 3003 genes and 2839 genes were identified as up- and down-regulated, respectively. The volcano plot shows the distribution of DEGs in GSE36295, GSE36693, and GSE65216, respectively, visualized by ipathwayguide (Figure 1A–C).

The intersection of gene expression changes among the three different GEO datasets was analyzed by FunRich software (Figure 1D). A total of 170 genes have been identified, showing the same direction of expression changes among three microarray data. 75 genes are up-regulated and 95 genes are down-regulated in TNBC patients compared with normal or other subtype breast cancer patients. Graphpad was used to show the expression pattern of intersection genes from three platforms as a heatmap. (1558 from GPL6244, 1854 from GPL10558 and 2467 from GPL570) (Figure 1E). These analyses provide gene expression changes across several TNBC databases.

### 3.2. Functional Enrichment Analysis of Overlapping DEGs

74 up-regulated and 94 down-regulated genes were identified as common in three datasets (Figure 1D). The gene enrichment analysis performed by FunRich is used to evaluate the function of identified genes. Gene enrichment analysis was used to gain mechanistic and functional insight into DEGs generated from genome-scale (omics) experiments. The function of DEGs was classified through the biological pathway; up-regulated DEGs were categorized into 263 groups and down-regulated DEGs were categorized into 251 groups (Table 3A). The result of the biological pathway shows that the up-regulated DEGs were mainly enriched in the categories of ‘Mitotic M-M/G1 phases’, ‘G2/M checkpoints’, ‘Cell cycle, mitotic’, ‘DNA replication’, ‘Cell cycle checkpoints’, ‘G2/M DNA damage checkpoint’, ‘Mitotic prometaphase’, ‘Activation of ATR in response to replication stress’, and ‘FOXM1 transcription factor network’. Down-regulated DEGs were significantly enriched in the category of ‘Epithelial-to-mesenchymal transition (EMT)’ pathway (Table 4A). In the study of cell component analysis, up-regulated DEGs were categorized into 63 groups and down-regulated DEGs were categorized into 48 groups (Table 3B). The result shows that up-regulated DEGs were mainly enriched in categories of ‘Chromosome’, ‘Nucleoplasm’, and ‘Chromosome passenger complex’, while down-regulated DEGs were significantly enriched in categories of ‘Extracellular’, ‘Extracellular space’, and ‘Extracellular matrix’ (Table 4B). In the study of cell component analysis, up-regulated DEGs were categorized into 35 groups and down-regulated DEGs were categorized into 41 groups (Table 4C). Down-regulated DEGs were significantly enriched in categories of ‘Extracellular matrix structural constituent’ and ‘Carboxypeptidase activity’, but up-regulated DEGs have no significant result.

### 3.3. PPI Network Analysis

**Figure 2:**
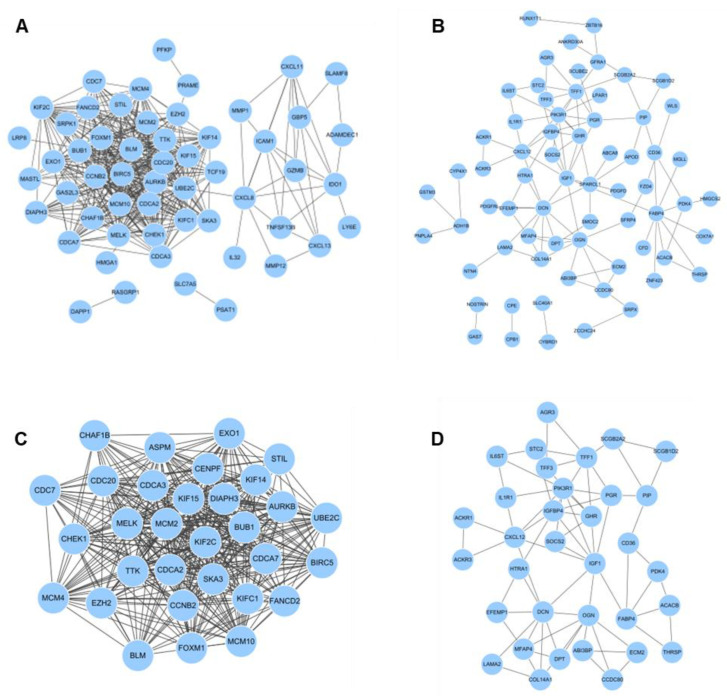
PPI network of DEGs. (**A**) PPI network of up-regulated and (**B**) down-regulated genes created by the STRING site. (**C**) Clustering analysis of up-regulated DEGs and (**D**) down-regulated DEGs for selecting hub genes using the MCODE plug-in in cytoscape.

To describe the function of identified genes from up- and down-regulated DEGs, the protein-protein interaction (PPI) network of genes was predicted with the STRING database. Up-regulated DEGs of 74 nodes and 444 edges were presented in PPI network (Figure 2A) and down-regulated DEGs of 94 nodes and 106 edges were presented in PPI network (Figure 2B) with all PPI enrichment *p* values < 1.0e-16. Subsequently, the MDOCE tool revealed up-regulated and down-regulated modules. The up-regulated module has 31 nodes and 381 edges (score = 25.4) and the down-regulated module has 34 nodes and 70 edges (score = 4.242) (Figure 2C,D). In addition, according to the results of the KEGG pathway analysis, up-regulated DEGs were significantly enriched in categories of ‘Cell cycle’ and ‘Rheumatoid arthritis’ (Table 5A). Down-regulated DEGs were significantly enriched in categories of ‘Pathways in cancer’ and ‘Regulation of lipolysis in adipocytes’ (Table 5B). As a consequence, 13 genes were found to be correlated with gene enrichment analysis and PPI network analysis in total DEGs as hub genes. Seven genes are part of up-regulated DEGs (MCM2, CDC20, BUB1, MCM4, CDC7, CCNB2, and CHEK1) and six genes are part of down-regulated DEGs (IGF1, CXCL12, IL6ST, and LMAM2). The above findings described the function and interaction of meaningfully altered gene expression in TNBC.

### 3.4. Survival Correlation Analysis

**Figure 3:**
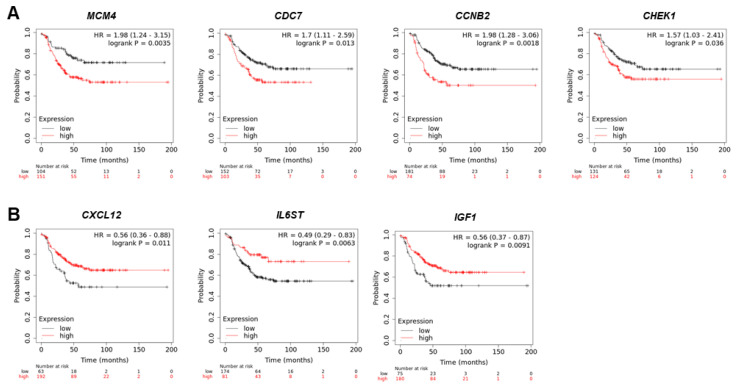
Survival rate analysis between TNBC patients and up-regulated and down-regulated genes. (**A**) The correlation of survival rate and up-regulated MCM4, CDC7, CCNB2, and CHEK1 gene expression in TNBC patients. (**B**) The correlation of survival rate and down-regulated CXCL12, IL6ST, and IGF1 gene expression in TNBC patients.

We analyzed the correlation of patient survival rates between TNBC patients and 13 gene candidates using the KM plotter website. In ER-, PR-, and HER2-status negative groups, the survival rates of 255 patients with each gene were evaluated. As a result, MCM4, CDC7, CCNB2, and CHEK1 genes with increased expression in TNBC show poor survival rates (Figure 3A). Conversely, CXCL12, IL6ST, and IGF1 with reduced expression in TNBC was found to be correlated with high survival rates (Figure 3B). Other genes showed no correlation with survival rates in TNBC patients (Figure S1). These findings suggest that the 7 genes are related to the survival rate of TNBC patients and are an independent prognostic indicator that can effectively predict the prognosis of TNBC patients.

### 3.5. Comparison of the DEGs Expression in Patients and Cells

**Figure 4:**
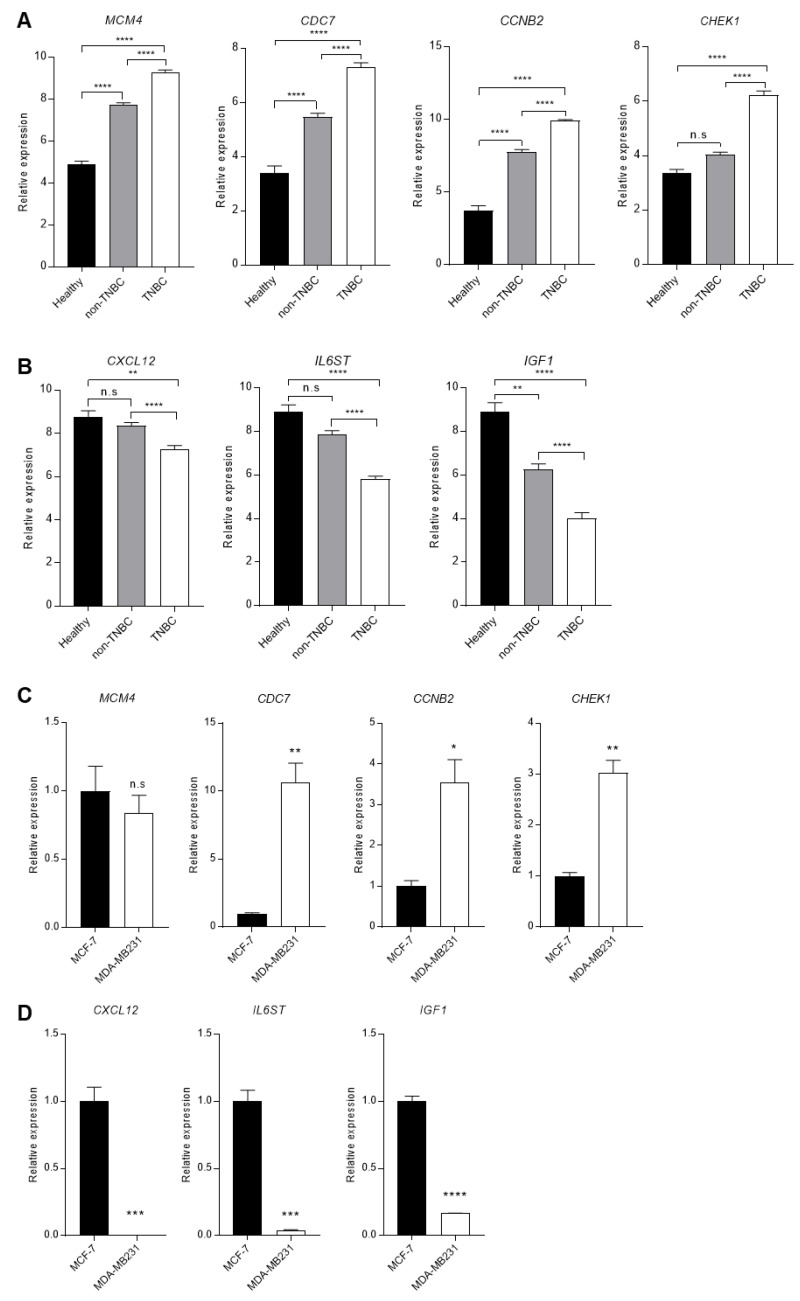
Comparison of DEGs expression between non-TNBC and TNBC patients or cell lines. (**A**) The comparison of up-regulated DEGs, MCM4, CDC7, CCNB2, and CHEK1, in Healthy, non-TNBC, and TNBC from GSE65216. (**B**) The comparison of down-regulated DEGs, CXCL12, IL6ST, and IGF1, in Healthy, non-TNBC, and TNBC. (**C**) The mRNA expression comparison of up-regulated DEGs, MCM4, CDC7, CCNB2, and CHEK1, in non-TNBC cells and TNBC cells, which are MCF-7 and MDA-MB231. (**D**) The mRNA expression comparison of down-regulated DEGs, CXCL12, IL6ST, and IGF1, in non-TNBC cells and TNBC cells, which are MCF-7 and MDA-MB231. Columns are presented with the mean of SEM. Statistical analysis using one-way ANOVA was performed in (**A**,**B)**, and *t*-test performed in (**C**,**D**); * *p* < 0.05, ** *p* < 0.005, *** *p* < 0.0005, **** *p* < 0.0001 vs. control in each group, n.s.: nonsignificant.

To confirm the expression difference of the 7 genes screened through survival rates, additional expression comparison analysis was performed on GSE65216, including healthy people, non-TNBC, and TNBC patients’ data. The 4 up-regulated DEGs were increased in TNBC patients compared with healthy and non-TNBC (Figure 4A). The expression of the 3 down-regulated DEGs was decreased in TNBC patients compared with healthy and non-TNBC (Figure 4B). The expression of the CHEK1, CXCL12, and IL6ST genes changed only in TNBC, and there was no significant change between healthy and non-TNBC.

Next, we checked the expression of up- and down-regulated DEGs in two breast cancer cell lines: non-TNBC cancer cell MCF-7 and TNBC cancer cell MDA-MB231. CDC7, CCNB2, and CHEK1 genes were increased in MDA-MB231 cells compared with MCF-7 cells (Figure 4C). However, the MCM4 showed no significant difference in the two cell lines. In the case of down-regulated genes, it was confirmed that all 3 genes were reduced in MDA-MB231 cells versus MCF-7 cells (Figure 4D). These results show that DEGs identified from the GSE database are differently expressed in TNBC patients and breast cancer cells.

### 3.6. Difference of CHK1 Protein Expression in Patients and Cells

**Figure 5:**
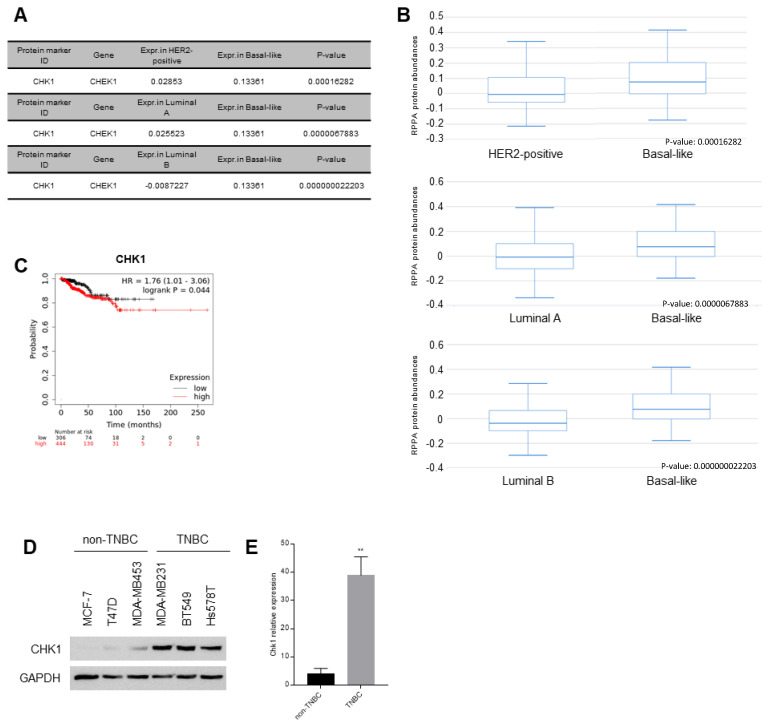
The expression of CHK1 protein according to breast cancer subtypes. (**A**) CHK1 protein expression level with basal-like subtype of breast patients versus HER2-positive, luminal A, and luminal B from ‘The cancer proteome atlas’. (**B**) CHK1 protein expression comparison graph from ‘The cancer proteome atlas’. (**C**) The survival rate of breast cancer patients according to CHK1 protein expression using the KM plotter website. (**D**) Western blotting of CHK1 in non-TNBC cell lines, including MCF-7 and T47D, and TNBC cell lines, including MDA-MB453, MDA-MB231, BT549, and Hs578T. (**E**) The graph showing quantification of CHK1 expression normalized with endogenous GAPDH through Graphpad. Columns are presented with the mean of SEM. Statistical analysis using *t*-test was performed by comparing non-TNBC and TNBC; ** *p* < 0.005 vs. control in each group.

Pathologically, breast cancer is classified into luminal A, luminal B, HER2-positive, and basal-like breast cancer. Although basal-like breast cancer and TNBC do not have exactly the same meaning, these are commonly used interchangeably because 77% of basal-like patients are TNBC and the prognosis of the two patients is similar [47]. Therefore, we conducted an analysis using a pathological classification database from ‘The cancer proteome atlas’ to find proteins with an increase or decrease in expression among the 7 genes. Only CHK1, a protein encoded by CHEK1, was overexpressed in basal-like breast cancer subtype versus HER2-positive, luminal A, and luminal B (Figure 5A,B). We analyzed the survival rate according to CHK1 protein expression using the KM plotter website, and found a graph with a decreasing survival rate of TNBC patients (Figure 5C). To confirm CHK1 protein expression changes, we checked the expression of CHK1 in TNBC and non-TNBC cell lines; as a result, increased expression was observed in TNBC cell lines compared with non-TNBC cell lines (Figure 5D,E).

**Figure 6:**
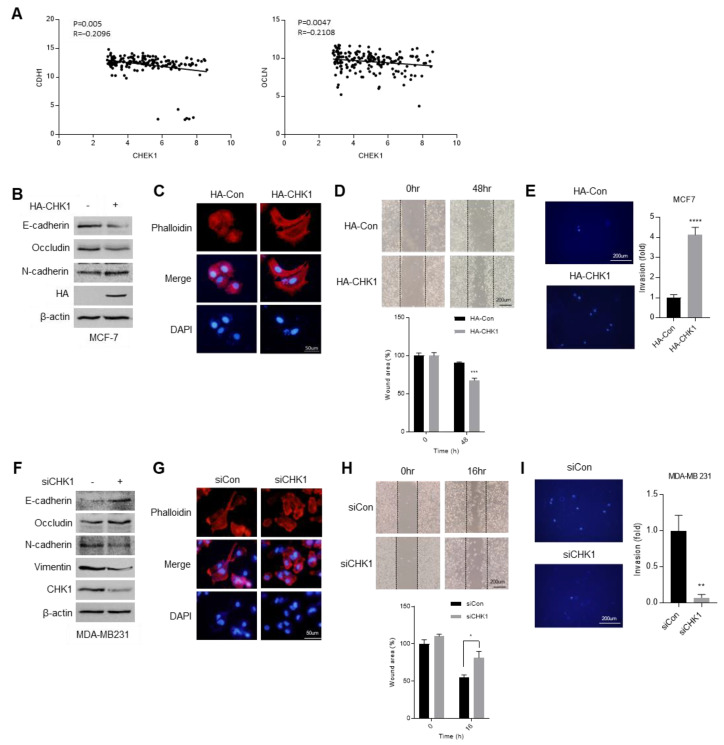
CHK1 induced epithelial to mesenchymal transition (EMT) in breast cancer cells. (**A**) The correlation of CHK1 with CDH1 and OCLN, which are epithelial marker genes. (**B**) Western blotting of EMT marker proteins in control and CHK1-overexpressing MCF-7 cells. (**C**) Fluorescence of phalloidin for observation of the morphology in control and CHK1-overexpression MCF-7 cells. (**D**) Migration assay with control and CHK1-overexpression MCF-7 cells for 48 h after scratch. (**E**) Transwell invasion assay with control and CHK1-overexpression MCF-7 cells for 48 h after incubation. (**F**) Western blotting of EMT marker proteins in control and CHK1-knockdown MDA-MB231 cells. (**G**) Fluorescence of phalloidin for observation of the morphology in control and CHK1-knockdown MDA-MB231 cells. (**H**) Migration assay with control and CHK1-knockdown MDA-MB231 cells for 16 h after scratch. (**I**) Transwell invasion assay with control and CHK1-knockdown MDA-MB231 cells for 12 h after incubation. Columns are presented with the mean of SEM. Statistical analysis using *t*-test was performed by comparing HA-Con and HA-CHK1 or siCon and siCHK1; * *p* < 0.05, ** *p* < 0.005, *** *p* < 0.0005, **** *p* < 0.0001 vs. control in each group.

The epithelial to mesenchymal transition (EMT) is the most representative phenomenon of malignant tumors [38]. Therefore, we investigated the correlation between CHEK1 gene and epithelial marker genes, which are CDH1 and OCLN, from the GSE 65216 dataset (Figure 6A). We found a negative correlation between CHEK1 and epithelial marker genes. Since the above results imply that CHEK1 may affect EMT in breast cancer, we confirmed EMT marker protein expression in non-TNBC MCF-7 cells versus HA-CHK1 (hemagglutinin tagged CHK1) overexpressing MCF-7 cells (Figure 6B). The epithelial markers, e-cadherin and occludin, were decreased and the mesenchymal marker, n-cadherin, was increased in CHK1-overexpressing MCF-7 cells. With the overexpression of CHK1, the cell morphology turned more malignant (Figure 6C), and the cell migration and invasion activity increased (Figure 6D,E). Furthermore, the epithelial markers, e-cadherin and occludin, were induced and the mesenchymal marker, n-cadherin, was reduced in CHK1-knockdown MDA-MA231 cells (Figure 6F). The CHK1 silencing cells showed more epithelial morphology compared with control MDA-MB231 cells (Figure 6G). Additionally, the migration and invasion activity decreased in CHK1-reduced cells (Figure 6H,I). Together, these results propose a new role for CHK1 as an EMT regulator; furthermore CHK1 can be utilized as a new diagnostic and treatment target for TNBC patients.

## 4. Discussion

Breast cancer has been well-classified by the presence or absence of three receptors, ER, PR, and HER2. TNBC is a type in which all three receptors are not expressed and is considered to be more aggressive than other subtypes and has higher mortality and recurrence rates [48]. There are two main reasons for poor prognosis: late diagnosis and lack of therapy target. Mortality rates of breast cancer patients are decreasing due to the advancement of receptor-targeted treatments; however, TNBC is difficult to diagnose and treat because it does not have receptors. Therefore, genetic analysis studies are required for the development of diagnostic markers and treatment targets for TNBC patients.

In this study, we found up- or down-regulated genes in TNBC across three GEO databases (GSE36295, GSE36693, and GSE65216) intersection analysis. In the three datasets, the genes that change expression were cut-off and screened based on FC log > 1 and *p*-value < 0.01, and the intersection between them were identified. Next, these genes were categorized as a biological pathway, cellular component, and molecular function, as well as KEGG pathway. We further obtained overlapping genes, 4 up-regulated genes, which are MCM4, CDC7, CCNB2, and CHEK1, and 3 down-regulated genes, which are CXCL12, IL6ST, and IGF1, from the correlation between gene enrichment analysis and PPI network analysis. These genes showed the correlation between gene expression and the survival rate of breast cancer patients. Subsequently, we investigated the expression level of 7 genes followed by breast cancer patient subtypes and breast cancer cell lines.

TNBC is being classified in more detail through gene expression analysis [9]. It is typically classified into basal-like (BL1 and BL2), a mesenchymal (M), and a luminal androgenreceptor (LAR) type, with different prognosis depending on the subtype [49]. Although there are differences according to TNBC subtypes, TNBC and basal-like breast cancer generally have the worst prognosis among each classification. Approximately 77% of TNBCs are overlapped basal-like, so they are often referred to as almost similar classifications [47]. Therefore, we compared based on basal-like breast cancer to observe the differences in the selected DEGs in the protein expression database according to pathological classification. Through proteome atlas, only the expression of CHK1, the protein of the CHEK1 gene, was uniquely enhanced in basal-like breast cancer patients compared with other subtype patients. The expression of CHK1 was upregulated in TNBC cell lines compared with non-TNBC cells.

In general, CHK1 reacts to DNA damage and is activated by phosphorylation [19]. CHK1 regulates various steps of the cell cycle including S, M phase, and G2/M transition and CHK1 has also been reported to have an increased expression in various cancers [24]. Cell cycle control genes are the main targets of cancer cell therapy. TP53 and BRCA1/2, which are known as representative genes mutated in breast cancer, are also known to affect cell cycle and cell proliferation [50,51]. In the case of TP53, several reports have announced that it is associated with the patient’s overall survival or risk of metastasis [52]. However, other studies have reported that this gene is not significantly related to the prognosis of patients, but has value as a potential marker [53]. Mutation of the BRCA1/2 gene is found not only in the breast but also in ovarian and pancreatic tumors, and this mutation occupied about 50% of breast tumor patients [54]. BRCA1 acts a majority in DNA repair, so this gene is important for tumorigenesis and tumor progression [55]. However, although TNBC tends to be diagnosed later than other cancers, the above two genes are unclear regarding the correlation of tumor metastasis, which is a main cause of mortality of high-grade tumor patients [16]. In addition, the mutation in PI3K pathway factors and tyrosine receptors is also observed, but it is difficult to say that this is a specific characteristic of TNBC; in fact, the mutation of these genes in TNBC patients is not higher than those in the above genes [56]. These reports suggest that the two well-known genes are insufficient to be applied as an apparent diagnostic marker and treatment target for breast cancer, and therefore persistent gene discovery is necessary.

Metastasis refers to a phenomenon in which cancer cells, converted from the primary tumor to the mesenchymal type, move through blood vessels and secondary tumors occur in other organs. In this process, the turnover of epithelial cells into mesenchymal cells used for metastasis is called EMT [57] and this state is essential for metastasis [58]. In this study, we identified that CHK1 is a potential molecule that can regulate EMT in breast cancer cells. CHK1 expression is related to the EMT marker genes and we demonstrate that it regulated expression of EMT marker proteins using gain or loss of function analysis. In addition, CHK1 increased mesenchymal phenotype, migration, and invasion activity in breast cancer cells. These findings imply that CHK1 plays a significant role in breast cancer metastasis and that it might be exploited as a diagnostic marker and a potential therapeutic target.

## 5. Conclusions

We explored the GSE database to find DEGs between TNBC and non-TNBC patients. Through biology enrichment and PPI network analysis, 13 genes were selected. Only 7 genes had a correlation with a survival rate of TNBC patients and the mRNA level was consistent with the screening results. Among the 7 genes, one gene was sorted through protein Atlas to find key factors affecting TNBC patients. CHK1 is known to be a major factor in controlling the cell cycle and is known to influence cancer cell growth. However, there is a lack of research on its association with metastasis, an important characteristic of malignant carcinoma such as TNBC. In this study, we demonstrated that the metastasis of cancer cells was increased by CHK1 through EMT marker level, cell phenotype, migration, and invasion activity assay. These results provide evidence that CHK1, found through the GSE database and several data analyses, can regulate metastatic ability through EMT in breast cancer cells.

## Figures and Tables

**Table 1:** Primer sequence used in RT-PCR.

Name of Gene	Direction	Nucleotide Sequence	Reference
MCM4	Forward Reverse	5′-GGCAGACACCACACACAGTT-3′ 5′-CGAATAGGCACAGCTCGATA-3′	[38]
CDC7	Forward Reverse	5′-TCAAACACCTCCAGGACAATAC-3′ 5′-GTACCTCATTCCAGCCTTCTAAA-3′	[39]
CCNB2	Forward Reverse	5′-AAAGCTCAGAACACCAAAGTTCCA-3′ 5′-ACAGAAGCAGTAGGTTTCAGTTGT-3′	[40]
CHEK1	Forward Reverse	5′-GGTCACAGGAGAGAAGGAAT-3′ 5′-TCTCTGACCATCTGGTTCAGG-3′	[41]
CXCL12	Forward Reverse	5′-ATGAACGCCAAGGTCGTGGTCG-3′ 5′-TGTTGTTGTTCTTCAGCCG-3′	[42]
IL6ST	Forward Reverse	5′-TGTAGATGGCGGTGATGGTA-3′ 5′-CCCTCAGTACCTGGACCAAA-3′	[43]
RUNX1T1	Forward Reverse	5′-ACGAACAGCTGCTTCTGGAT-3′ 5′-TGCTTGGATGTTCTGAGTGC-3′	[44]
IGF1	Forward Reverse	5′-CCATGTCCTCCTCGCATCTC-3′ 5′-TTGAGGGGTGCGCAATACAT-3′	[45,46]

**Table 2:** Microarray datasets of TNBC employed in present study.

GEO Series	TNBC Samples	Non-TNBC Samples	Platform	Reference
GSE 36295	11	39	Affymetrix Human Gene 1.0 ST Array	Merdad A et al. Anticancer Res (2014)
GSE 36693	21	66	Illumina HumanHT-12 V4.0 expression beadchip	Lee ST et al. Proc Natl Acad Sci USA (2013)
GSE 65216	53	100	Affymetrix Human Genome U133 Plus 2.0 Array	Maire V et al. Cancer Res (2013)

**Table 3:** Enrichment analysis of up-regulated DEGs. (**A**) Biological pathway. (**B**) Cellular component.

(**A**)					
**Biological Pathway**	**No. of Genes in the Dataset**	**Percentage of Genes**	**Fold Enrichment**	***p*-Value (Bonferroni Method)**	**Genes Mapped (from Input Data Set)**
Mitotic M-M/G1 phases	11	27.5	7.152153	0.000313	MCM2; CDC20; AURKB; MCM4; CDC7; BIRC5; KIF2C; MCM10; UBE2C; BUB1; CENPF;
2/M Checkpoints	6	15	21.96786	0.000383	MCM2; MCM4; CDC7; MCM10; CCNB2; CHEK1;
Cell Cycle, Mitotic	12	30	5.955975	0.000568	MCM2; CDC20; AURKB; MCM4; CDC7; BIRC5; KIF2C; MCM10; CCNB2; UBE2C; BUB1; CENPF;
DNA Replication	11	27.5	6.631518	0.000672	MCM2; CDC20; AURKB; MCM4; CDC7; BIRC5; KIF2C; MCM10; UBE2C; BUB1; CENPF;
Cell Cycle Checkpoints	8	20	10.67079	0.000939	MCM2; CDC20; MCM4; CDC7; MCM10; CCNB2; CHEK1; UBE2C;
G2/M DNA damage checkpoint	5	12.5	27.15018	0.001423	MCM2; MCM4; CDC7; MCM10; CHEK1;
Mitotic Prometaphase	7	17.5	11.13068	0.003929	CDC20; AURKB; BIRC5; KIF2C; UBE2C; BUB1; CENPF;
Activation of ATR in response to replication stress	5	12.5	21.28146	0.005033	MCM2; MCM4; CDC7; MCM10; CHEK1;
FOXM1 transcription factor network	5	12.5	18.74856	0.009595	AURKB; BIRC5; FOXM1; CCNB2; CENPF;
(**B**)					
**Cellular Component**	**No. of Genes in the Dataset**	**Percentage of Genes**	**Fold Enrichment**	***p*** **-Value (Bonferroni Method)**	**Genes Mapped (from Input Data Set)**
Chromosome	5	7.462687	18.4384887	0.005756	MCM2; AURKB; MCM4; BIRC5; BUB1;
Nucleoplasm	10	14.92537	4.84162639	0.028453	CHAF1B; MCM2; CDC20; MCM4; CDC7; FANCD2; WDR4; MCM10; CHEK1; UBE2C;
Chromosome passenger complex	2	2.985075	145.024966	0.048965	AURKB; BIRC5;

**Table 4:** Enrichment analysis of down-regulated DEGs. (**A**) Biological pathway. (**B**) Cellular component. (**C**) Molecular function.

(**A**)					
**Biological Pathway**	**No. of Genes in the Dataset**	**Percentage of Genes**	**Fold Enrichment**	***p*-Value (Bonferroni Method)**	**Genes Mapped (from Input Data Set)**
Epithelial-to-mesenchymal transition	17	31.48148	10.70746	1.34E-10	SFRP4; ECM2; DCN; SPARCL1; CXCL12; F13A1; COL14A1; LHFP; ZCCHC24; RUNX1T1; AKAP12; EFEMP1; DPT; SRPX; JAM2; MFAP4; IGF1;
(**B**)					
**Cellular Component**	**No. of Genes in the Dataset**	**Percentage of Genes**	**Fold Enrichment**	***p*-Value (Bonferroni Method)**	**genes MAPPED (from Input Data Set)**
Extracellular	36	39.56044	3.15516144	5.15E-08	SCGB2A2; SCGB1D2; TFF1; TFF3; HTRA1; AGR3; SFRP4; PIP; ECM2; CPB1; LAMA2; IGFBP4; NTN4; CCDC80; DCN; SPARCL1; CXCL12; SMOC2; SCUBE2; IL6ST; F13A1; STC2; COL14A1; PDGFD; SEPP1; APOD; SEMA3C; GHR; EFEMP1; DPT; SRPX; OGN; CFD; MFAP4; IGF1; ZBTB16;
Extracellular space	13	14.28571	5.1493083	0.001012	SCGB1D2; ABI3BP; HTRA1; SFRP4; DCN; IL6ST; COL14A1; APOD; GHR; EFEMP1; DPT; OGN; IGF1;
Extracellular matrix	8	8.791209	10.6728104	0.000636	ABI3BP; HTRA1; DCN; COL14A1; EFEMP1; DPT; OGN; MFAP4;
(**C**)					
**Molecular Function**	**No. of Genes in the Dataset**	**Percentage of Genes**	**Fold Enrichment**	***p*-Value (Bonferroni Method)**	**Genes Mapped (from Input Data Set)**
Extracellular matrix structural constituent	8	8.510638	9.30256	0.000534	ECM2; LAMA2; NTN4; DCN; COL14A1; EFEMP1; DPT; MFAP4;
Carboxypeptidase activity	3	3.191489	27.62133	0.037609	CPB1; CPA3; CPE;

**Table 5:** KEGG pathway analysis of total DEGs. (**A**) Up-regulated DEGs. (**B**) Down-regulated DEGs.

(**A**)				
**KEGG Pathways**	**No. of Genes in the Dataset**	**Percentage of Genes**	***p*-Value (Bonferroni Method)**	**Genes Mapped (from Input Data Set)**
Cell cycle	8	10.81081	3.22e-06	TTK; MCM2; CDC20; BUB1; CDC7; CHEK1; MCM4; CCNB2
Rheumatoid arthritis	5	6.75675	0.0011	HLA-DOB; TNFSF13B; ICAM1; CXCL8; MMP1
(**B**)				
**KEGG Pathways**	**No. of Genes in the Dataset**	**Percentage of Genes**	***p*-Value (Bonferroni Method)**	**Genes Mapped (from Input Data Set)**
Pathways in cancer	10	10.63829	0.026	FZD4; IGF1; PIK3R1; CXCL12; IL6ST; ZBTB16; RUNX1T1; LPAR1; LAMA2; GSTM3
Regulation of lipolysis in adipocytes	4	4.25532	0.026	XGLL; FABP4; PIK3R1; PLA2G16

## Data Availability

The GSE datasets gained by GEO profiles section in https://www.ncbi.nlm.nih.gov (accessed on 29 January 2021).

## Supplementary Materials

The following supporting information can be downloaded at: https://www.mdpi.com/article/10.3390/cimb44120398/s1, Figure S1: Uncorrelated survival rate between TNBC patients and up-regulated and down-regulated genes.
