# Supplementary material for: Checkpoint Kinase 1 (CHK1) Functions as Both a Diagnostic Marker and a Regulator of Epithelial-to-Mesenchymal Transition (EMT) in Triple-Negative Breast Cancer
Source: Curr Issues Mol Biol. 2022 Nov 23;44(12):5848–65. doi: 10.3390/cimb44120398 (PMC9777496; doi:10.3390/cimb44120398)
Supplement: Supplementary file 1 [file cimb-44-00398-s001.zip › cimb-2023267-supplementary.pdf]

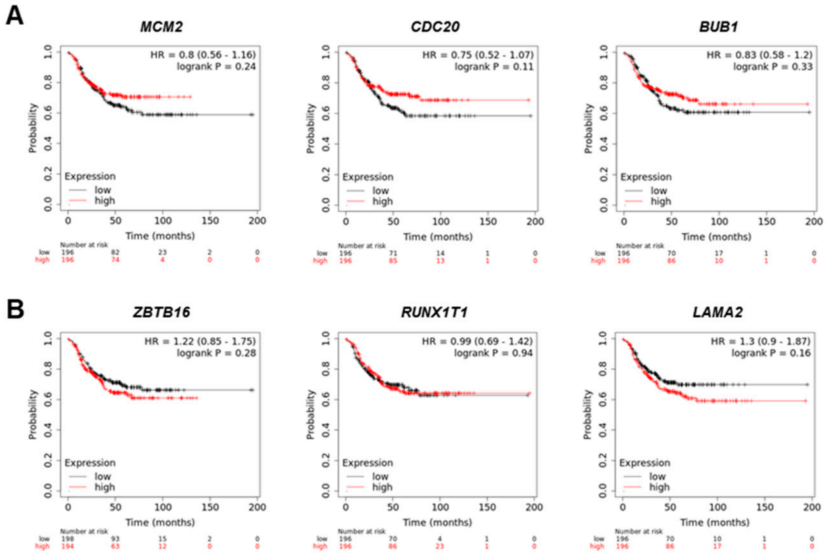

Supplementary Figure S1. Uncorrelated survival rate between TNBC patients and up-regulated and down-regulated genes. (A) The correlation of survival rate and up-regulated MCM2, CDC20 and BUB1 gene expression in TNBC patients. (B) The correlation of survival rate and down-regulated ZBTB16, RUNX1T1 and LAMA2 gene expression in TNBC patients.
